# The Gut Microbiome as a Biomarker and Therapeutic Target of Immune Checkpoint Inhibitors: A Review for Oncologists

**DOI:** 10.3390/cells14221779

**Published:** 2025-11-12

**Authors:** Thiti Susiriwatananont, Panuch Eiamprapaporn, Maria Vazquez Roque, Francis A. Farraye, Adam Perlman, Saranya Chumsri

**Affiliations:** 1 Division of Medical Oncology, Department of Medicine, Faculty of Medicine, Chulalongkorn University and The King Chulalongkorn Memorial Hospital, Bangkok 10330, Thailand; thiti.su@chula.ac.th; 2 Jacoby Center for Breast Health, Division of Hematology and Medical Oncology, Mayo Clinic, Jacksonville, FL 32224, USA; 3 Department of Medicine, Faculty of Medicine, Thammasat University Hospital, Pathum Thani 12120, Thailand; 4 Inflammatory Bowel Disease Center, Division of Gastroenterology and Hepatology, Mayo Clinic, Jacksonville, FL 32224, USA; 5 Pendulum Therapeutics, Inc., San Francisco, CA 94110, USA

**Keywords:** immune checkpoint inhibitors, gut microbiome, cancer immunotherapy, biomarkers, multi-omics integration, microbial diversity, fecal microbiota transplantation, probiotics/prebiotics/postbiotics

## Abstract

Immune checkpoint inhibitors (ICIs) have transformed cancer therapy, yet their benefits remain limited to a subset of patients, underscoring the need for more reliable biomarkers and novel therapeutic strategies. The gut microbiome has emerged as a critical modulator of systemic immunity and a promising determinant of ICI response. Evidence links specific microbial features, taxa, and bioactive metabolites to enhanced antitumor immunity, whereas disruptions, such as antibiotic exposure, are associated with poorer outcomes. Advances in sequencing and multi-omics technologies have provided more profound insights into microbiome-immune crosstalk, though methodological heterogeneity continues to challenge reproducibility. Translational studies demonstrate that microbiome-based intervention, including fecal microbiota transplantation (FMT), biotics supplementation, and engineered microbial strains, can enhance ICI efficacy or mitigate immune-related toxicities. Despite encouraging early clinical signals, broader implementation requires methodological rigor, standardized protocols, and innovative trial designs that account for host and environmental factors. For clinicians, the most immediate strategies involve prudent antibiotic stewardship and patient enrollment in microbiome-focused clinical trials. Overall, the gut microbiome is a promising biomarker and a therapeutic target, representing a new frontier for personalizing immunotherapy and improving patient outcomes in oncology.

## 1. Introduction

The approval of immune checkpoint inhibitors (ICIs) in the 2010s marked a paradigm shift in cancer therapy. Used as monotherapy or in combination, ICIs have produced unprecedented outcomes and are now the standard of care across several solid tumors. However, a substantial proportion of patients derive limited benefit, and efficacy can vary even among patients with the same cancer type and comparable biomarkers [1,2].

In parallel, the gut microbiome has emerged as a key regulator of host immunity. Accumulating evidence suggests that the microbiome may serve as both a predictive biomarker of ICI response and as a therapeutic target to enhance efficacy. Incorporating microbiota-based strategies into ICI therapy, therefore, represents a promising approach to improving clinical outcomes [3].

This review aims to provide practicing medical oncologists with a practical overview of how the gut microbiome affects antitumor immunity and influences the efficacy of ICIs. The following sections outline the limitations of current ICI biomarkers and describe how the microbiome is acquired and interacts with the host immune system. The discussion then covers the analytical methods used to study gut microbiota in relation to ICI response, microbial features associated with treatment outcomes, and therapeutic strategies targeting the gut microbiome. The review concludes by highlighting key challenges that future research must address for successful clinical application.

## 2. Current Biomarkers of ICIs and Their Limitations

ICIs have delivered long-term, durable responses in metastatic cancer and increased cure rates in early-stage disease. Despite this success, it is estimated that only ~13% of cancer patients are eligible and derive benefit from ICI therapy [4]. Currently, several biomarkers are used in clinical practice to guide ICI use, including programmed death-ligand 1 (PD-L1) expression, mismatch repair (MMR) or microsatellite instability (MSI) status, and tumor mutational burden (TMB). Other promising markers include POLE/POLD1 mutation and tumor-infiltrating lymphocytes (TILs). Despite extensive validation across tumor types, no single biomarker reliably predicts response to ICIs in all settings. Their performance is context-dependent and limited by assay heterogeneity, tumor evolution, and sampling bias. A summary of their methods, advantages, and limitations is presented in Table 1.

The limitations of these current ICI biomarkers justify the search for integrative models that incorporate host factors, including the gut microbiome, to better predict treatment outcomes.

## 3. The Microbiome: A Key Environmental Factor in Immunity

The human immune responses vary considerably across individuals, shaped by a combination of intrinsic host factors and external environmental influences. Host factors such as genetic background, age, and sex are known to affect immune cell composition and antitumor surveillance [14,15,16]. However, evidence from twin studies suggests that environmental factors, including the gut microbiome, infections, and medications (notably antibiotics and immunomodulatory drugs), are the dominant drivers, accounting for 24–77% of the variability in human immune traits [17,18].

### 3.1. Acquisition and Distribution

The gut microbiome is highly individualized down at the strain level [19,20], with environmental factors dominating over genetics in shaping its composition in adults [21,22,23]. Microbial colonization begins at birth via maternal transmission and evolves throughout life in response to interactions with other humans and animals, diet, antibiotic exposure, and the surrounding environment [24]. Microbial communities are found throughout the gastrointestinal tract, but their composition varies significantly by location due to differences in oxygen levels, pH, and transit time [25,26]. The large intestine hosts the most abundant and diverse community, making it the primary focus for biomarker discovery and therapeutic modulation.

### 3.2. How the Gut Microbiome Modulates Antitumor Immunity

**Figure 1:**
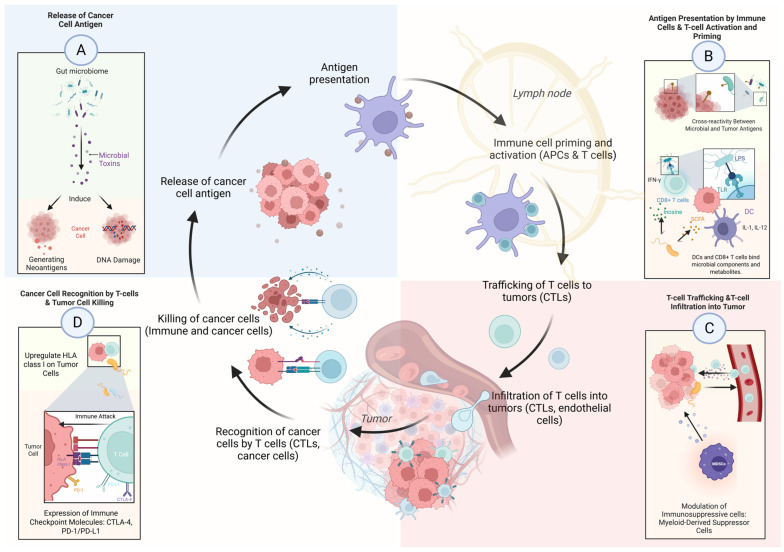
Examples of microbiome interaction across the cancer-immunity cycle. The gut microbiome modulates antitumor immunity through multiple steps of the cancer–immunity cycle. (**A**) Microbial components and toxins can induce DNA damage and generate neoantigens. (**B**) Microbial antigens cross-react with tumor antigens and stimulate dendritic cell maturation via Toll-like receptor (TLR) signaling, enhancing antigen presentation and T-cell priming. (**C**) Microbiota-derived metabolites and cytokines regulate chemokine expression and myeloid-derived suppressor cell (MDSC) activity, facilitating T-cell trafficking and infiltration into the tumor microenvironment. (**D**) Within tumors, microbial signals can enhance HLA class I expression and modulate immune checkpoint molecules, promoting T-cell–mediated tumor killing. Abbreviations: CTL, cytotoxic T lymphocyte; DC, dendritic cell; IFN, interferon; IL, interleukin. Created with BioRender.com/0bwtrk8 (accessed on 9 November 2025).

The gastrointestinal tract houses one of the body’s largest immune compartments [27]. The gut microbiome modulates both innate and adaptive immune responses through two primary mechanisms: direct stimulation via microbial components (e.g., lipopolysaccharides activating Toll-like receptors) and indirect signaling via production of bioactive metabolites (e.g., short-chain fatty acids and secondary bile acids). Table 2 summarizes key microbiome-mediated mechanisms that affect both innate and adaptive immunity, including interactions across the cancer-immunity cycle. Figure 1 presents an integrated schematic of how these microbial processes influence antigen release, immune activation, T-cell trafficking, and tumor cell killing.

The gut microbiome is not a passive bystander but an active modulator of systemic immunity, with the potential to either enhance or suppress antitumor immune responses. Its ability to influence every step of the cancer-immunity cycle makes it a powerful biomarker and a promising therapeutic target in oncology.

## 4. Analysis of Gut Microbiome and Response to ICIs

### 4.1. Analysis Pipeline Overview

**Figure 2:**
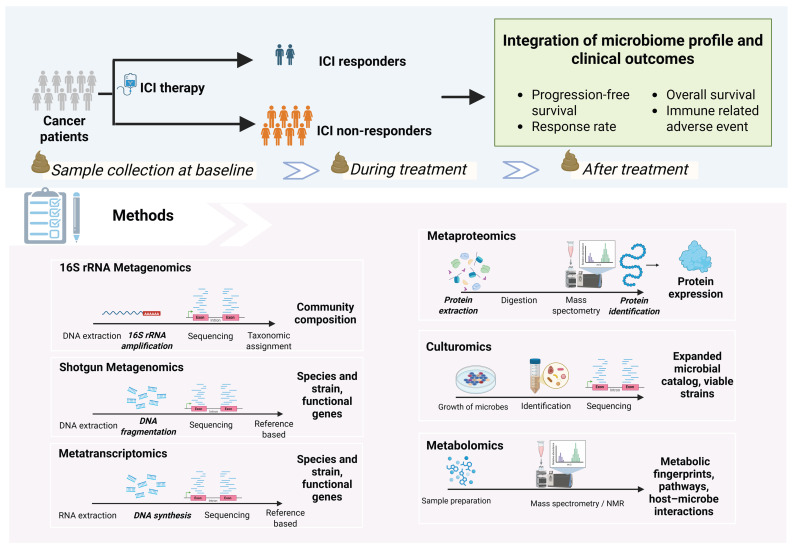
Analysis pipeline of gut microbiome and ICI response. Schematic of the study design and analytical workflow for investigating the gut microbiome in ICI-treated patients. Stool samples are typically collected at baseline, during, and after ICI therapy for responders and non-responders. Integrative analyses link microbiome profiles to clinical outcomes, including progression-free survival, overall survival, and immune-related adverse events. Lower panels illustrate commonly used analytical approaches: 16S rRNA and shotgun metagenomics for taxonomic and functional profiling; metatranscriptomics, metaproteomics, and metabolomics for gene expression, protein, and metabolite analysis; and culturomics for isolating viable strains and expanding microbial catalogs. Created with BioRender.com/9bguul0 (accessed on 9 November 2025).

The investigation of the gut microbiome and ICI response follows a structured pipeline: (1) prospective sample collection at key time points; (2) stratification of patients into ICI responder and non-responder groups; (3) microbiome profiling and bioinformatic processing; and (4) statistical analysis to integrate microbiome data with clinical outcomes (Figure 2).

Reproducibility is a major challenge in microbiome research. Discrepancies between studies often arise from variations at each step of the pipeline. Adherence to reporting standards, such as the Minimum Information about a Marker Gene Sequence (MIMARKS), and detailed documentation of protocols are crucial for enabling cross-study comparisons and validating findings [50].

### 4.2. Sample Types for Microbiome Profiling

#### 4.2.1. Fecal Samples

Fecal specimens are currently the gold standard for gut microbiome analysis, serving as a proxy for the distal colon’s microbial community. Collection is non-invasive, repeatable, and provides sufficient biomass for analysis [51]. To preserve microbial integrity, samples should be immediately cryopreserved at −80 °C or stored in commercial preservation buffers. Standardized protocols for collection, storage, and transport are essential, as variability can significantly alter results [52,53].

#### 4.2.2. Oral Samples

Oral samples (e.g., saliva and swabs) are readily accessible, but their role in systemic immune modulation is less well established [54]. These samples are also limited by low microbial biomass, high levels of human DNA contamination, and significant variability across oral sites [55,56].

#### 4.2.3. Direct Gut Sampling (Swab, Biopsy, and Swallowable Capsules)

Mucosal biopsy and luminal swab enable direct study of host tissue and microbiome interactions. Catheter aspiration facilitates sampling from difficult-to-reach sites. However, these methods are invasive, requiring special preparation, and may not be suitable for routine use. Swallowable capsules can autonomously collect intestinal fluid at the targeted gut location with minimal invasiveness. Nevertheless, their adoption is limited by technical complexity and availability [51].

#### 4.2.4. Tumor Samples

Historically considered sterile, tumor tissue is now recognized to harbor distinct microbial communities, referred to as the intratumoral microbiome. Early hints date back to the 1890s with William B. Coley’s work on bacterial toxin-mediated antitumor effects [57], and modern sequencing technologies have since confirmed the presence of these microbes. Intratumoral microorganisms are now considered integral components of the tumor microenvironment and have been linked to treatment outcomes, representing a promising new frontier of investigation [58,59,60,61,62,63].

### 4.3. Relative vs. Absolute Quantification of Microbiome

Microbiome data can be interpreted in two ways:

- Relative abundance: This is the default output of standard sequencing that measures the proportion of each microbe within a sample (e.g., *Bacteroides* make up 20% of the community). While widely used, this approach is prone to compositionality bias—an increase in one taxon will automatically appear as a decrease in others, even if their absolute numbers remain unchanged [64,65].
- Absolute abundance: This measures the actual number or concentration of microbes (e.g., 10^9^ CFU/g of *Lactobacillus*). It mitigates compositional bias by integrating sequencing data with complementary quantitative techniques, such as quantitative PCR (qPCR), flow cytometry, or the addition of synthetic spike-in standards (reference DNA or microbes added in known quantities for calibration), providing a more accurate measurement of microbial load, which is essential for robust biological interpretation [66,67].

Relying on relative abundance alone can lead to false correlations [68,69]. For example, after probiotic administration, an increase in the relative abundance of *Lactobacillus* may reflect a decline in other commensals rather than true colonization. In contrast, detecting *Lactobacillus* at levels of 10^9^ CFU/g via absolute quantification confirms successful engraftment and intervention efficacy. Absolute quantification is crucial for developing robust and reliable biomarkers.

### 4.4. Methods for Microbiome Analysis

#### 4.4.1. Sequencing-Based Methods

- 16S rRNA gene sequencing: This cost-effective method targets the 16S rRNA gene, a universal “barcode” present in all bacteria. The 16S rRNA gene contains conserved regions that serve as universal primer binding sites and hypervariable regions that enable species-specific taxonomic classification [70]. It provides a broad overview of community composition, typically at the genus level. While excellent for assessing overall diversity, its lower resolution makes species- or strain-level identification challenging [71,72]. Recent advances in full-length 16S rRNA sequencing have improved the taxonomic resolution of this technique [73,74].
- Shotgun metagenomics: This technique sequences all genomic DNA in a sample, providing a high-resolution view of the community at the species and strain level. It can also identify fungal, viral, archaeal, and protozoan communities [75]. Additionally, this approach enables the inference of the functional and metabolic potential of microbial communities at the gene level. However, precise identification of novel functional genes may be limited by the availability and comprehensiveness of reference databases [76,77].
- Metatranscriptomics (RNA-Seq): This method analyzes RNA to reveal which microbial genes are actively expressed. It offers a dynamic snapshot of the microbiome’s actual functional activity, but it is technically challenging due to RNA’s instability and complex data analysis [78,79]

#### 4.4.2. Culture- and Metabolic-Based Methods

- Culturomics: While sequencing identifies microbes by their genetic code, culturomics aims to grow them in the laboratory. By using diverse culture conditions, this technique allows for the isolation of live strains, including rare or novel bacteria that may be missed by traditional methods [80]. Culturing microbes enables functional experiments and the development of next-generation probiotics [81,82]. However, microbial culturing is labor-intensive, costly, requires advanced infrastructure, and carries a risk of contamination [83].
- Metabolomics: This approach identifies and quantifies the small-molecule metabolites produced by the host and microbiome using mass spectrometry (MS) or nuclear magnetic resonance (NMR) [84]. Linking metagenomic data (the community’s genetic potential) with metabolomic data (its actual chemical output) can provide deep mechanistic insights [85]. However, untargeted metabolomics has some limitations, including difficulty in accurately identifying many metabolites and interference from matrix effects such as ion suppression, which can affect measurement accuracy and make comparisons between studies challenging [86,87].

#### 4.4.3. Multi-Omics Integration: A Holistic View

The most robust insights now emerge from multi-omics approaches that integrate metagenomics, metatranscriptomics, proteomics, and metabolomics. This holistic strategy can unravel complex host-microbe interactions and identify robust, multifaceted biomarkers. While challenges in standardization and data complexity remain, multi-omics integration is essential for translating microbiome discoveries into reliable clinical applications.

## 5. Microbial Features Associated with ICI Response

Although reproducibility can be a challenge in microbiome research, several key microbial features have been consistently identified as predictors of ICI efficacy. These signatures represent promising biomarkers and therapeutic targets.

### 5.1. Microbial Diversity

Microbial diversity is assessed in two main ways:

- Alpha diversity: The richness (number of different organisms present) and evenness (their relative abundance) within a single sample.
- Beta diversity: The degree of compositional difference between samples.

In multiple cancer types, higher baseline alpha diversity has been associated with improved responses to ICI therapy [88,89,90,91]. However, the magnitude of this difference is generally modest and often overlaps between groups. A meta-analysis reported that greater microbial diversity correlated with improved PFS (HR = 0.64, 95% CI 0.42–0.98), although heterogeneity across studies was high [92]. Conversely, reduced diversity, often observed in patients exposed to antibiotics, has been linked to poorer clinical outcomes [88,93].

### 5.2. Beneficial Bacterial Taxa

Most studies implemented 16S rRNA sequencing or shotgun metagenomics to measure relative abundance. As noted in Section 4.3, this compositional approach limits interpretation, and absolute quantification will be essential in future studies to validate these associations. Given these methodological limitations, several bacterial taxa have been linked to enhanced ICI response:

#### 5.2.1. *Akkermansia muciniphila*

*A. muciniphila* is a common human gut microbe, specializing in degrading mucin and maintaining intestinal integrity [94]. In preclinical models, oral administration of *A. muciniphila* can reinvigorate exhausted T cells and restore anti-PD1 efficacy [95]. Higher relative abundance of *A. muciniphila* in stool at baseline is associated with improved progression-free survival in non-small cell lung cancer (NSCLC) and renal cell carcinoma (RCC) treated with PD-1 blockade [96,97,98]. In a prospective study of 338 advanced NSCLC patients, baseline stool *A. muciniphila* was associated with increased objective response rates and overall survival, independent of PD-L1 status [99].

#### 5.2.2. *Faecalibacterium prausnitzii*

*F. prausnitzii*, a major butyrate producer, is one of the most abundant bacteria in gut microbiota, representing more than 5% of gut microbiota in healthy adults [100,101]. In patients diagnosed with cancer, the relative abundance of *F. prausnitzii* was significantly lower than in non-cancer subjects [102,103]. In vitro, the *F. prausnitzii* strain EXL01 enhances dendritic cell and T cell activity, and oral administration of this strain restores anti-PD-L1 efficacy in mouse models with antibiotic-induced microbiota disruption [104]. Enrichment of *F. prausnitzii* at baseline has been observed in ICI responders across multiple cancer types [89,105,106].

#### 5.2.3. *Bifidobacterium* Species

*Bifidobacteria* are predominant in infants but generally constitute a smaller proportion (<10% of the relative abundance) of the adult gut microbiota [107]. In mouse models, oral administration of *Bifidobacterium* enhances ICI effects by augmenting dendritic cell function and producing the metabolite inosine [40,108,109,110]. Enrichment of *B. longum* and *B. breve* has been associated with improved outcomes in melanoma and NSCLC patients receiving ICIs, respectively [111,112].

#### 5.2.4. *Ruminococcaceae* Family

Members of the Ruminococcaceae family are highly prevalent in the human gut microbiome, with particular species having been linked to ICI response [113]. For example, *R. gnavus* enhanced anti–PD-1 efficacy in mouse models by promoting CD4^+^ T cell migration into tumors and activating pro-inflammatory macrophages [114]. In clinical cohorts of advanced NSCLC and melanoma patients, the Ruminococcaceae family was enriched in ICI responding patients [88,115]

### 5.3. Key Microbial Metabolites

#### 5.3.1. Short-Chain Fatty Acids (SCFAs)

SCFAs—notably butyrate, acetate, and propionate—are produced by the gut microbiota through fermentation of dietary fiber. They play critical roles in linking the microbiome to both local and systemic immune regulation. These functions are mediated by several key mechanisms, including histone deacetylase (HDAC) inhibition, G-protein-coupled receptor (GPCR) signaling, and modulation of cellular energy and signaling pathways [116]. However, clinical data are mixed: some studies link high fecal and plasma SCFA levels to better ICI outcomes [117,118,119], while others find no significant correlation [120,121,122]. Such discrepancies may reflect context-dependent effects of SCFAs on regulatory and effector T cells within the tumor microenvironment [38,123].

#### 5.3.2. Inosine

Inosine, a purine nucleoside, is involved in purine metabolism, RNA function, and immune modulation [124]. Produced by bacteria such as *Bifidobacterium pseudolongum*, inosine activates antitumor T cells through adenosine A2A receptor signaling in preclinical models [40]. In RCC patients, higher plasma inosine levels were associated with response to nivolumab [125].

#### 5.3.3. Tryptophan Metabolites

The essential amino acid tryptophan can be metabolized through multiple pathways that influence both cancer progression and antitumor immunity, including the kynurenine, indole-3-pyruvate, and serotonin pathways. Tumor cells frequently channel tryptophan into the immunosuppressive kynurenine pathway, whereas commensal gut microbes can redirect it toward alternative routes. For example, gut bacteria can convert tryptophan into a ligand for the aryl hydrocarbon receptor (AHR), a key regulator of intestinal homeostasis [126]. Certain metabolites, such as indole-3-aldehyde (I3A) produced by *Lactobacillus reuteri*, enhance CD8^+^ T cell activity in mouse models [127]. In cancer patients, plasma biomarkers, including the kynurenine-to-tryptophan (Kyn/Trp) ratio and metabolites such as I3A and 3-hydroxyanthranilic acid (3-HAA), have been correlated with ICI efficacy [127,128,129].

#### 5.3.4. Secondary Bile Acids

In addition to their role in lipid absorption, bile acids function as systemic signaling molecules regulating host metabolism and immune function [130]. Primary bile acids are synthesized in the liver and subsequently converted by microbial enzymes, mainly from *Clostridium* species such as *Clostridium butyricum*, into secondary bile acids. These secondary bile acids display stronger activation of host nuclear receptors than their primary counterparts [131]. In hepatocellular carcinoma (HCC) models, accumulation of conjugated bile acids in tumors impairs anti-PD-1 efficacy, whereas dietary supplementation of ursodeoxycholic acid (UDCA) suppresses tumor growth and promotes T cell responses [132]. Clinically, HCC patients receiving ICIs who had fecal enrichment of UDCA and ursocholic acid demonstrated improved treatment outcomes [133].

### 5.4. The Importance of Temporal Dynamics

The gut microbiome is not static; its composition shifts dynamically following ICI treatment, with distinct patterns emerging between responders and non-responders. Notably, certain microbial alterations emerge only after treatment initiation, rather than at baseline.

In melanoma and NSCLC patients receiving ICI therapy, responders exhibit stable microbial taxa and functional profiles over time compared to non-responders [134,135]. Other studies have shown that both responders and non-responders develop unique microbial abundance patterns after ICI initiation, even when baseline differences are minimal or absent [90,136,137].

### 5.5. Tools Incorporating Microbial Signature to Predict Prognosis and ICI Response

Emerging tools are being developed to integrate microbial signatures into prognostic models and ICI response prediction. Although still in early stages and requiring further validation, these approaches demonstrate the translational potential of microbiome-informed biomarkers.

- TOPOSCORE: Developed from metagenomic data of 245 NSCLC patient feces combined with *Akkermansia* quantification, TOPOSCORE is a qPCR-based assay targeting 21 bacteria to evaluate personal intestinal dysbiosis. Validated in NSCLC, colorectal cancer, genitourinary cancer, and melanoma patients, TOPOSCORE was able to stratify patients with improved ICI outcomes. The test can be performed within 48 h, making it potentially suitable for routine clinical practice [138].
- miCRoScore: miCRoScore is a composite multi-omics biomarker developed from the microbiome and immune gene signature of 348 colon cancer patients. It outperforms conventional prognostic biomarkers in colon cancer, including Consensus Molecular Subtypes (CMS) and microsatellite instability, in predicting survival probability. Patients classified with high mICRoScore showed an excellent 97% 5-year overall survival in the training cohort, with no colon cancer-related deaths observed in the external validation cohort [139].

As a biomarker, the gut microbiome offers both predictive and prognostic potential in immuno-oncology. Specific microbial signatures and functional profiles have been linked to ICI response, resistance, and toxicity, suggesting that microbiome-based metrics could complement existing biomarkers. However, it is important to note that although several studies report similar microbiome signals, the findings remain inconclusive, as not all studies consistently replicate the same results. Ongoing efforts to standardize studies and improve analytical pipelines in microbiome research are expected to enhance reproducibility and reliability.

## 6. Therapeutic Applications of the Gut Microbiome

**Figure 3:**
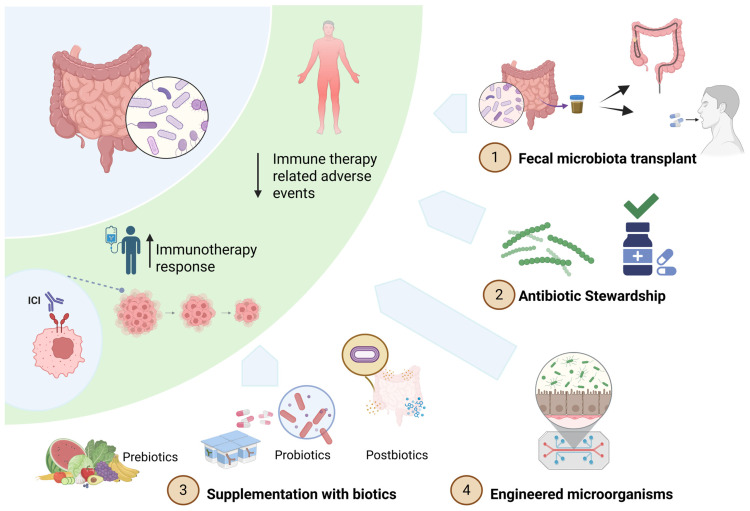
Therapeutic applications of the gut microbiome to enhance ICI therapy. The gut microbiome can influence both efficacy and toxicity of immune checkpoint inhibitors (ICIs). Therapeutic strategies to optimize microbiome composition include: (1) Fecal microbiota transplantation (FMT): transfer of a healthy or responder-derived microbial community; (2) Antibiotic stewardship: minimizing microbiome-disruptive antibiotic use; (3) Biotic supplementation: administration of prebiotics, probiotics, or postbiotics to restore beneficial microbial function; and (4) Engineered microorganisms: synthetic or genetically modified bacteria designed to deliver immunomodulatory molecules. These interventions aim to enhance ICI response and reduce immune-related adverse events. Created with BioRender.com/goujhtp (accessed on 9 November 2025).

The early evidence supporting the therapeutic potential of the gut microbiome in oncology came from preclinical studies in which fecal microbiota transplantation (FMT) from ICI-responsive patients into germ-free mice enhanced antitumor immune responses, whereas FMT from non-responders did not [96,112,115]. These findings were later translated into early-phase clinical trials. Subsequent proof-of-concept clinical studies in ICI-refractory melanoma patients confirmed that FMT, when combined with re-induction anti-PD1, could overcome ICI resistance in a subgroup of patients by altering the gut microbiome and tumor microenvironment [140,141]. Building on these findings, several strategies are now under investigation to modulate the microbiota to enhance immunotherapy efficacy and mitigate treatment-related toxicity. These include FMT, supplementation of pre-, pro-, and postbiotics (collectively referred to as biotics), engineered microbial strains, and antibiotic modulation (Figure 3).

### 6.1. Fecal Microbiota Transplant (FMT) to Enhance ICI Efficacy

Early-phase studies of FMT combined with anti-PD1 re-challenge in ICI-refractory melanoma showed encouraging signals, with overall response rates (ORR) of 20–30% and durable disease control exceeding 6–12 months in responders. Responders of FMT typically demonstrated greater donor microbiome engraftment and enrichment of specific bacterial taxa. More recent studies have expanded FMT evaluation to earlier-line settings and across multiple tumor types (Table 3a,b).

FMT was generally safe and feasible across studies, with most adverse events related to mild gastrointestinal symptoms (grade 1–2). Although donor-derived infection is a recognized potential risk of FMT [123], it has not yet been reported in studies combining FMT with ICI therapy. However, the FMT-LUMINate melanoma cohort reported a notably high incidence (65%) of ≥grade 3 irAEs, likely reflecting the additive toxicity of dual ICI therapy and potential donor-related immune effects, as 43% of participants received FMT from the same donor. This finding underscores that FMT is not without risk and highlights the critical importance of donor selection and stringent safety monitoring in future trial designs.

Despite promising efficacy signals, FMT protocols have shown considerable heterogeneity, including donor selection (responder-derived versus healthy individuals), use of different preconditioning regimens (antibiotics and bowel preparation), route of administration (oral or colonoscopy), and maintenance dosing. Further research is needed to clarify cancer-specific and regimen-dependent differences in microbiome–immune interactions.

Higher microbial engraftment has consistently been associated with improved clinical outcomes. Identification of species-specific engraftment patterns and integrating metagenomic analyses may enable personalized FMT strategies [149,150,151]. Ongoing efforts aim to define the optimal donor characteristics, preparation, and delivery methods to maximize therapeutic benefit.

### 6.2. Fecal Microbiota Transplant (FMT) to Mitigate ICI-Induced Colitis

Colitis is the most extensively studied irAE in relation to gut microbiome composition. Reduced microbiome diversity and prior antibiotic use have been linked to the development of ICI-related colitis [152,153,154,155]. Shifts in microbial composition, particularly with strain-specific enrichment, have also been observed; however, the results are inconsistent [156,157,158,159]. Administration of *F. prausnitzii* and *Bifidobacterium* helps mitigate ICI-related colitis in animal models [160,161]. Several case reports have shown improvement of steroid-refractory immune-related colitis following FMT administration [162,163]. Ongoing clinical trials are exploring the benefits of FMT in preventing (Clinical Trials.gov identifiers: NCT04163289 and NCT06508034) and treating immune-related colitis in patients receiving ICIs (Clinical Trials.gov identifiers: NCT04038619, NCT06206707, and NCT06499896). Preliminary data from a prospective study suggest that frontline FMT is safe and can be an effective treatment, reducing the need for corticosteroids [164].

### 6.3. Supplementation with Biotics: A Targeted Approach

Unlike FMT, which introduces the entire microbial community and environment, biotic interventions such as prebiotics, probiotics, and postbiotics provide a more targeted approach to transform the gut microbiome and host immune system. According to the International Scientific Association for Probiotics and Prebiotics (ISAPP), the definitions of prebiotics, probiotics, and postbiotics are summarized in Table 4.

Traditional probiotics mainly include a limited number of species, such as *Lactobacillus* and *Bifidobacterium*, which have a long history of safe use as food ingredients or dietary supplements. Advances in microbiology techniques and bioinformatics have enabled the precise identification and culture of novel gut microbes with therapeutic potential. These next-generation probiotics (NGPs) include *Prevotella copri*, *Christensenella minuta*, *Parabacteroides goldsteinii*, *Akkermansia muciniphila*, *Bacteroides thetaiotaomicron*, *Faecalibacterium prausnitzii*, *Bacteroides fragilis*, and *Eubacterium hallii* [168,169]. Notably, *A. muciniphila* and *F. prausnitzii* have shown potential to enhance ICI efficacy in preclinical and translational studies, as mentioned above.

Several phase 1 studies have explored the benefits of adding probiotics in combination with ICIs in advanced RCC patients (Table 5).

Although studies employing *Clostridium butyricum* CBM588—a butyrate-producing bacterial strain—did not increase the abundance of beneficial *Bifidobacterium* spp., the observed improvement in clinical outcomes suggests that CBM588 may exert its effects through alternative mechanisms, such as modulation of microbial metabolites or enrichment of other taxa like *Ruminococcaceae*, which have been associated with favorable ICI responses [171].

Despite promising mechanistic insights, clinical data on the use of biotics to enhance immunotherapy in cancer patients remain in the early stages. Current challenges include heterogeneity and a lack of standardized quality control for biotic products, interference from patients’ background diets, and limited trial designs that account for microbiome variability. Therefore, no firm recommendations on routine use of prebiotics, probiotics, or postbiotics in ICI-treated patients can be made at this time. Several ongoing trials are now investigating dietary interventions and biotic supplementation as adjuncts to ICIs across multiple cancer types, aiming to establish whether microbiome-targeted strategies can improve response rates and reduce immune-related toxicities [173].

### 6.4. Engineered Microorganisms

Advances in genetic and synthetic biology have enabled the development of engineered microorganisms that can augment immunotherapy against cancer in multiple aspects. By modifying microbial chassis, researchers can add features that promote immune activation while minimizing systemic side effects associated with naturally derived microbes (Table 6).

Beyond antigen delivery and cytokine release, engineered strains can also remodel the tumor microenvironment and deliver immunomodulatory payloads directly into the microenvironment. However, challenges include limited tumor colonization in humans, competition with endogenous microbiota, potential systemic inflammation, and reproducibility [177,178].

### 6.5. Antibiotic Stewardship

Inappropriate antibiotic use can disrupt intestinal homeostasis and cause dysbiosis characterized by reduced microbial diversity, expansion of potentially harmful strains, and depletion of beneficial taxa. Intestinal dysbiosis has been associated with impaired efficacy of ICIs. While randomized controlled trials studying this association are not feasible, extensive preclinical data and multiple large observational studies and meta-analyses [179,180] consistently demonstrate that antibiotic exposure around the time of ICI initiation (up to 90 days before and after) is linked to inferior clinical outcomes.

Current recommendations emphasize strict adherence to international antibiotic stewardship principles [181,182]. Clinicians should confirm the presence of infection before initiating antibiotics, prioritize narrow-spectrum agents when possible, prescribe the shortest effective duration, and regularly assess the indication. These measures help minimize the negative impact of antibiotics on ICI efficacy.

## 7. Conclusions and Outlook

The clinical translation of gut microbiome research in immuno-oncology depends on overcoming key challenges of standardization and validation. Future progress will require:

- Methodological rigor: implementation of absolute quantification, multi-omics integration, and standardized protocols for sample collection, processing, and analysis
- Innovative trial designs: prospective studies that incorporate dietary profile, antibiotic exposure, and dynamic microbial signatures, with interval stool sampling and predefined microbiome-specific endpoint
- Refined interventions: development of optimized microbial consortia, inclusion of next-generation biotics, standardized reporting of biotic composition and FMT protocols, and rational FMT donor selection within a robust safety framework to enable scalability.

The relationship between the gut microbiome and cancer immunotherapy has evolved from a compelling association to an emerging field with growing clinical relevance and tangible translational potential. Looking forward, integrating microbiome-derived biomarkers with established predictors such as PD-L1 and TMB may enable the development of composite stratification tools, improving patient selection and optimizing immunotherapy outcomes. For practicing medical oncologists, the most immediate and impactful strategies remain prudent antibiotic stewardship and encouraging patient enrollment in microbiome-focused clinical trials.

In summary, the gut microbiome is both a powerful biomarker and a therapeutic target that can shape immunotherapy outcomes. Continued methodological refinement, innovative clinical approaches, and translational research will be critical to realizing its full potential and bringing microbiome-informed strategies into routine oncology practice.

## Figures and Tables

**Table 1:** Current biomarkers for immune checkpoint inhibitors.

Biomarker	Methods	Advantages	Limitations
Programmed death-ligand 1 (PD-L1) [5]	immunohistochemistry (IHC)	widely available; quick turnaround; validated in several cancer types	multiple FDA-approved companion assays (22C3, 28-8, SP142, SP263) with different score cut-off for ICIs and cancer types; subject to tumor heterogeneity and sampling bias
Mismatch repair (MMR) [6,7,8]	PCR or next-generation sequencing (NGS) for MSI status; IHC for MMR proteins (MLH1, PMS2, MSH2, MSH6)	FDA-approved MSI-high (MSI-H) or dMMR status as a tissue-agnostic biomarker; strong predictive value	rare in solid tumors (~3–16%); limited availability of validated MSI assays in some centers
Tumor mutational burden (TMB) [9,10]	NGS	FDA-approved TMB ≥ 10 mut/Mb by FoundationOne CDx as a tissue-agnostic biomarker for pembrolizumab; reflects overall neoantigen landscape	expensive and longer turnaround time; optimal cut-off may vary across cancer types; lack of standardized assessment methods
POLE/POLD1 mutations [11,12]	NGS	associated with an ultra-hypermutated phenotype and exceptionally high TMB	not FDA-approved; rare in solid tumors (~4%)
Tumor-infiltrating lymphocytes (TILs) [13]	H&E pathology slide evaluation	reflects the actual immune response within the tumor; assessable on routine pathology slides	not FDA-approved; lack standardized scoring; subject to spatial and temporal heterogeneity

**Table 2:** Examples of Mechanisms of Microbiome-Immune Crosstalk.

Innate Immunity	
- Maintenance of intestinal mucosal integrity and homeostasis through production of mucins and metabolites [28]. - Bacterial components (e.g., LPS and flagellin) activate pattern recognition receptors (e.g., TLRs), promoting maturation of dendritic cells (DCs), polarization of M1 macrophages, and activation of NK cells [29]. - Bacterial metabolites enhance the function of innate lymphoid cells [30].	
**Examples of microbiome interactions across the cancer-immunity cycle**	
Release of cancer cell antigens	- Microbial toxins can induce DNA damage, generating neoantigens [31] - The microbiota can influence tumor-associated antigens presentation and immunogenic cell death [32]
Antigen Presentation by Immune Cells & T-cell Activation and Priming	- Cross-reactivity between microbial and tumor antigens [33,34] - Promotes DC activation and maturation via TLR signaling [35] - Enhanced cytokine production (e.g., IL-1, IL-12) by dendritic cells [36] - Microbiota-derived SCFAs (butyrate and propionate) modulate DC function and T-cell differentiation by histone deacetylases (HDAC) inhibition [37,38,39] - Microbiota-derived inosine boosts IFN-γ production in CD8+T cells via adenosine receptor (A2A) signaling [40]
T-cell Trafficking & T-cell Infiltration into the Tumor	- Regulation of chemokines and cytokines influencing T-cell trafficking (e.g., TNF-α, CXCL9, CXCL10) [41,42] - Modulation of immunosuppressive cells like Myeloid-Derived Suppressor Cells (MDSCs) [43,44]
Cancer Cell Recognition by T cells & Tumor Cell Killing	- Influences the expression of immune checkpoint molecules (e.g., CTLA-4, PD-1/PD-L1) [45,46,47] - Can upregulate HLA class I on tumor cells, enhancing their recognition by T cells [48,49]

**Table 3:** (**a**): Selected clinical trials of FMT combined with ICIs in melanoma. (**b**): Selected clinical trials of FMT combined with ICIs in other solid tumors.

Study	N	Phase	Population	Intervention	Key Outcomes	≥Grade 3 irAEs
(**a**)						
Baruch et al. (2021) [140]	10	I	ICI-refractory melanoma	Responder-derived FMT + Nivolumab	ORR 30%; all responders with >6 mo PFS	0%
Davar et al. (2021) [141]	15	I	ICI-refractory melanoma	Responder-derived FMT + Pembrolizumab	ORR 20%; 3 patients with >12 mo stable disease	0%
MiMic (2023) [142,143]	20	II	Untreated meta-static melanoma	Healthy donor FMT + pembrolizumab or nivolumab	ORR 65%; median PFS 29.6 mo; median OS 52.8 mo	25%
FMT- LUMINate (2024) [144]	20	II	Untreated meta-static melanoma	Healthy donor FMT + anti-PD1 + anti-CTLA4	ORR 75%	65% (myocarditis 15%)
(**b**)						
Kim et al. (2024) [145]	13	I	ICI-refractory cancer (gastric n = 4, esophageal n = 5, HCC n = 4)	Responder-derived FMT + nivolumab	ORR 7.7%	7.7%
RENMIN-215 (2023) [146,147]	20	II	Refractory MSS colorectal cancer (≥2 prior lines)	Responder-derived FMT + Tislelizumab + Fruquintinib	ORR 20%; median PFS 9.6 mo; median OS 13.7 mo	10%
FMT- LUMINate (2024) [144]	20	II	Untreated meta-static NSCLC	Healthy donor FMT + anti-PD1	ORR 80%	0%
TACITO (2024) [148]	50	II	Untreated meta-static RCC	Intervention: Responder-derived FMT + pembrolizumab + axitinib Control: Placebo + pembrolizumab + axitinib	ORR 54% vs. 28% Median PFS 14.2 vs. 9.2 mo; 1-year PFS rate 66.7% vs. 35%; median OS NR vs. 25.3 mo	10%

**Table 4:** Definition of biotics according to ISAPP [165,166,167].

Biotic	Definition	Function	Examples
Prebiotics	substrates that are selectively utilized by host microorganisms, conferring a health benefit	nourish beneficial microbes, promoting their growth and metabolite production	Galactooligosaccharides (GOS), Fructooligosaccharides (FOS), Inulin, lactulose; naturally present in whole grains, onions, garlic, asparagus, bananas
Probiotics	live microorganisms that, when administered in adequate amounts, confer a health benefit.	directly introduce beneficial microbes to shape the gut environment	fermented foods such as yogurt, kefir, miso, natto, kimchi, and some cheeses containing specific live microbes (e.g., *Lactobacillus acidophilus*, *Bifidobacterium longum*)
Postbiotics *	preparation of inanimate microorganisms and/or their components that confer a health benefit.	deliver beneficial effects without living organisms, using inactivated microbial cells, components, or metabolites	heat-inactivated *Bifidobacterium* or *Lactobacillus*, bacterial lysates

* Per the ISAPP definition, purified metabolites alone, such as isolated butyric acid, do not meet the definition of postbiotic because they are not part of an inactivated microbial preparation.

**Table 5:** Selected phase 1 clinical trials of probiotics combination with ICIs in advanced RCC patients.

Study	N	Phase	Intervention	Primary Endpoint	Results and Safety
Dizman et al. (2022) [170]	30	I	Nivolumab + ipili-mumab ± CBM588 (*Clostridium butyricum*)	Change in *Bifidobacterium* spp. abundance at 12 weeks	No difference in *Bifidobacterium* spp. abundance. The CBM588 arm had a significantly improved PFS (12.7 vs. 2.5 mo) and ORR (58% vs. 20%). No significant difference in toxicity between the two arms.
Ebrahimi et al. (2024) [171]	30	I	Cabozantinib + nivolumab ± CBM588	Change in *Bifidobacterium* spp. abundance at 13 weeks	No difference in *Bifidobacterium* spp. abundance. The CBM588 arm had a significantly higher ORR (74% vs. 20%, *p* = 0.01). 6-mo PFS: 84% vs. 60%. No significant difference in toxicity between the two arms.
Derosa et al. (2025) [172]	9	I	Nivolumab + ipili-mumab + Onco-bax^®^-AK (*A. massiliensis* strain p2261, SGB9228) in patients lacking stool *Akkermansia*	ORR, pharmacodynamics, safety	ORR 50% with evidence of immune and metabolic modulation. No toxicity related to Onco-bax^®^-AK observed.

**Table 6:** Examples of early-phase clinical trials exploring engineered microorganisms in combination with ICIs.

Mechanisms	Examples
Presentation of tumor antigens and cancer vaccine carriers	Phase 2: ADXS-503, an engineered *Listeria monocytogenes* expressing 22 common NSCLC antigens, combined with pembrolizumab after progression. Induced antigen-specific T-cell responses with 15% ORR (2/13) [174]. Phase 2a: VXM01, an oral VEGFR2 DNA vaccine delivered via engineered *Salmonella Typhi* Ty21a, combined with avelumab in recurrent glioblastoma. Showed 12% ORR (3/25) [175].
Cytokine and chemokine release to enhance immune function	Phase 1: SYNB1891, engineered *E. coli* Nissle expressing a STING agonist, given intratumorally ± atezolizumab. Activated IFN pathways and immune gene signatures in refractory cancers [176].

## Data Availability

No new data were created or analyzed in this study.

## Abbreviations

The following abbreviations are used in this manuscript:

16S rRNA	16S ribosomal RNA
3-HAA	3-hydroxyanthranilic acid
A2A	Adenosine A2A receptor
AHR	Aryl hydrocarbon receptor
CFU	Colony-forming units (e.g., CFU/g)
CMS	Consensus Molecular Subtypes (colon cancer)
CTLA-4	Cytotoxic T-lymphocyte–associated protein 4
CXCL9/CXCL10	C-X-C motif chemokine ligand 9/10
DC/DCs	Dendritic cell(s)
dMMR	Deficient mismatch repair
DNA	Deoxyribonucleic acid
*E. coli*	*Escherichia coli*
FDA	U.S. Food and Drug Administration
FMT	Fecal microbiota transplantation
GPCR	G-protein–coupled receptor
H&E	Hematoxylin and eosin (stain)
HCC	Hepatocellular carcinoma
HDAC	Histone deacetylase
HLA	Human leukocyte antigen
ICI/ICIs	Immune checkpoint inhibitor(s)
IFN-γ	Interferon-gamma
IL-1/IL-12	Interleukin-1/Interleukin-12
IHC	Immunohistochemistry
I3A	Indole-3-aldehyde
irAE(s)	Immune-related adverse event(s)
Kyn/Trp	Kynurenine-to-tryptophan ratio
LPS	Lipopolysaccharide
MDSC(s)	Myeloid-derived suppressor cell(s)
MLH1, PMS2, MSH2, MSH6	Mismatch repair proteins/genes
MMR	Mismatch repair
MS	Mass spectrometry
MSI	Microsatellite instability
MSI-H	Microsatellite instability–high
NGP(s)	Next-generation probiotic(s)
NGS	Next-generation sequencing
NK (cells)	Natural killer (cells)
NMR	Nuclear magnetic resonance
NR	Not reached (survival endpoint)
NSCLC	Non-small-cell lung cancer
ORR	Objective response rate
OS	Overall survival
PCR	Polymerase chain reaction
PD-1	Programmed cell death protein 1
PD-L1	Programmed death-ligand 1
PFS	Progression-free survival
POLE/POLD1	DNA polymerase epsilon/delta 1 (genes)
qPCR	Quantitative PCR
RCC	Renal cell carcinoma
RNA-Seq	RNA sequencing (metatranscriptomics)
SCFA(s)	Short-chain fatty acid(s)
STING	Stimulator of interferon genes
TIL(s)	Tumor-infiltrating lymphocyte(s)
TLR(s)	Toll-like receptor(s)
TMB	Tumor mutational burden
TNF-α	Tumor necrosis factor-alpha
UDCA	Ursodeoxycholic acid
VEGFR2	Vascular endothelial growth factor receptor 2
